# Supplementary material for: Discovery and Characterization of a Novel Umbravirus from Paederia scandens Plants Showing Leaf Chlorosis and Yellowing Symptoms
Source: Viruses. 2022 Aug 19;14(8):1821. doi: 10.3390/v14081821 (PMC9414234; doi:10.3390/v14081821)
Supplement: Supplementary file 1 [file viruses-14-01821-s001.zip › viruses-1851844-supplementary.pdf]

Table S1. Primers used in this study.

Primer	Sequence 5′ > 3′
PSCYV-detection-F	CCAATGTTCAACTAGGTGGG
PSCYV-detection-R	ACTCTCTGCCGCTAATGTAT
PSCYV-5RACEGSP1-R	GTTGGCTGGAACCACATCGAGGTGGCCA
PSCYV-5RACEGSP2-R	GGGATGCTTCGTCGGGCTTTGGTGAAGC
PSCYV-3RACEGSP1-F	ACTGAAACCCCAAGCCGGAGGAACTGTT
PSCYV-3RACEGSP2-F	TCTTGCTGGTGTGGACTACCAGTGACCC
PSCYV-F	CGGGTATCGAAACCCCGATT
PSCYV-R	CGGGCGCGGTTGCAGTGATA
PSCYV-P1-2-F1	ATGGCGAGCGTTCTTGCCA
PSCYV-P1-2-R1	TCAAAAAATCCTTGGCCCCGGCCCCAA
PSCYV-P1-2-F2	CCAAGGATTTTTTGATTGGGGACGTCGT
PSCYV-P1-2-R2	CTAGCACACACGCCACAAC
PSCYV-P1-GFP-F	GAGCTCGGTACCCGGGGATCCATGGCGAGCGTTCTTGCCAA
PSCYV-P1-GFP-R	CATGTCGACTCTAGAGGATCCAAAATCCTTGGCCCCGGCCC
PSCYV-P2-GFP-F	GAGCTCGGTACCCGGGGATCCATGTTGTACAAGATGCTGGG
PSCYV-P2-GFP-R	CATGTCGACTCTAGAGGATCCGCACACACGCCACAACTCGC
PSCYV-P3-GFP-F	GAGCTCGGTACCCGGGGATCCATGGCCAGCGTTATCAATGT
PSCYV-P3-GFP-R	CATGTCGACTCTAGAGGATCCCCATTTGTTGGCAGTAATGC
PSCYV-P4-GFP-F	GAGCTCGGTACCCGGGGATCCATGTCTACCACACTCTCCAG
PSCYV-P4-GFP-R	CATGTCGACTCTAGAGGATCCGTAGCGTGGTCGTGATGGAC
PSCYV-P1-2-GFP-F	GAGCTCGGTACCCGGGGATCCATGGCGAGCGTTCTTGCCAA
PSCYV-P1-2-GFP-R	CATGTCGACTCTAGAGGATCCGCACACACGCCACAACTCGC
PSCYV-P1-Flag-F	GAGCTCGGTACCCGGGGATCCATGGCGAGCGTTCTTGCCA
PSCYV-P1-Flag-R	GTCGTCGACTCTAGAGGATCCAAAATCCTTGGCCCCGGCC
PSCYV-P2-Flag-F	GAGCTCGGTACCCGGGGATCCATGTTGTACAAGATGCTGGG
PSCYV-P2-Flag-R	GTCGTCGACTCTAGAGGATCCGCACACACGCCACAACTCG
PSCYV-P3-Flag -F	GAGCTCGGTACCCGGGGATCCATGGCCAGCGTTATCAATGT
PSCYV-P3-FLag-R	GTCGTCGACTCTAGAGGATCCCCATTTGTTGGCAGTAATGC
PSCYV-P4-Flag -F	GAGCTCGGTACCCGGGGATCCATGTCTACCACACTCTCCAG
PSCYV-P4-Flag-R	GTCGTCGACTCTAGAGGATCCGTAGCGTGGTCGTGATGGAC
PSCYV-P1-2-Flag-F	GAGCTCGGTACCCGGGGATCCATGGCGAGCGTTCTTGCCA
PSCYV-P1-2-Flag-R	GTCGTCGACTCTAGAGGATCCGCACACACGCCACAACTCG
PSCYV-PVX-P1-F	TCAGCACCAGCTAGCATCGATATGGCGAGCGTTCTTGCCA
PSCYV-PVX-P1-R	AACCGTTCATCGGCGGTCGACTCAAAAATCCTTGGCCCCGG
PSCYV-PVX-P2-F	AGCTAGCATCGATTGGCGCGCCATGTTGTACAAGATGCTGGG
PSCYV-PVX-P2-R	AACCGTTCATCGGCGGTCGACCTAGCACACACGCCACAAC
PSCYV-PVX-P3-F	AGCTAGCATCGATTGGCGCGCCATGGCCAGCGTTATCAATG
PSCYV-PVX-P3-R	AACCGTTCATCGGCGGTCGACTCACCATTTGTTGGCAGTAAT
PSCYV-PVX-P4-F	TCAGCACCAGCTAGCATCGATATGTCTACCACACTCTCCAG
PSCYV-PVX-P4-R	AACCGTTCATCGGCGGTCGACTTAGTAGCGTGGTCGTGATG
PSCYV-PVX-P1-2-F	TCAGCACCAGCTAGCATCGATATGGCGAGCGTTCTTGCCA

PSCYV-PVX-P1-2-R	AACCGTTCATCGGCGGTCGACCTAGCACACACGCCACAAC
PSCYV-pGD-P1-F	TACAAATCTATCTCTGGATCCATGGCGAGCGTTCTTGCCA
PSCYV-pGD-P1-R	GTTTGAACGAGCTCTGTCGACTCAAAAATCCTTGGCCCCG
PSCYV-pGD-P2-F	TACAAATCTATCTCTGGATCCATGTTGTACAAGATGCTGGG
PSCYV-pGD-P2-R	GTTTGAACGAGCTCTGTCGACCTAGCACACACGCCACAACT
PSCYV-pGD-P3-F	TACAAATCTATCTCTGGATCCATGGCCAGCGTTATCAATGT
PSCYV-pGD-P3-R	GTTTGAACGAGCTCTGTCGACTCACCATTTGTTGGCAGTAATG
PSCYV-pGD-P4-F	TACAAATCTATCTCTGGATCCATGTCTACCACACTCTCCAG
PSCYV-pGD-P4-R	GTTTGAACGAGCTCTGTCGACTTAGTAGCGTGGTCGTGATG
PSCYV-pGD-P1-2-F	TACAAATCTATCTCTGGATCCATGGCGAGCGTTCTTGCCAA
PSCYV-pGD-P1-2-R	GTTTGAACGAGCTCTGTCGACCTAGCACACACGCCACAACT
pCB301-PSCYV-F	AGTTCATTTCATTTGGAGAGGCGGGTATCGAAACCCCGATT
pCB301-PSCYV-R	GTGGAGATGCCATGCCGACCCGGGCGCGGTTGCAGTGATAA

Table S2. Viruses of the genus *Umbravirus* used for the nucleotide and amino acid identity analyses.

**Virus name**	**Nucleotide identities**	**Amino acids identities**				
	**Genome**	**P1**	**P2**	**RdRp**	**P3**	**P4**
Carrot mottle mimic virus	NC_001726.1	NP_054006.3	None	NP_054007.4	NP_054008.1	NP_054009.1
Carrot mottle virus	NC_011515.1	YP_009336474.1	None	YP_002302259.2	YP_002302260.1	YP_002302261.1
Ethiopian tobacco bushy top virus	NC_024808.1	YP_009336475.1	None	YP_009056849.1	YP_009056850.1	YP_009056851.1
Groundnut rosette virus	NC_003603.1	NP_619659.1	None	YP_009162058.1	NP_619660.1	NP_619661.1
Ixeridium yellow mottle virus 2	NC_034243.1	YP_009352230.1	None	YP_009352229.1	YP_009352231.1	YP_009352232.1
Opium poppy mosaic virus	NC_027710.2	YP_009164949.1	None	YP_009162614.2	YP_009162615.1	YP_009162616.1
Patrinia mild mottle virus	NC_055564.1	YP_010087618.1	None	YP_010087617.1	YP_010087619.1	YP_010087620.1
Pea enation mosaic virus 2	NC_003853.1	NP_620845.1	None	NP_620846.3	NP_620847.1	NP_620848.1
Tobacco bushy top virus	NC_004366.1	NP_733847.1	None	NP_733848.2	NP_733849.1	NP_733850.1
Tobacco mottle virus	NC_043206.1	None	None	YP_009665185.1	YP_009665186.1	YP_009665187.1

Table S3. Viruses used for phylogenetic analysis.

Virus name	RdRp Accession number	Genus
Carrot mottle mimic virus	NP_054007.4	*Umbravirus*
Carrot mottle virus	YP_002302259.2	*Umbravirus*
Ethiopian tobacco bushy top virus	YP_009056849.1	*Umbravirus*
Groundnut rosette virus	YP_009162058.1	*Umbravirus*
Ixeridium yellow mottle virus 2	YP_009352229.1	*Umbravirus*
Opium poppy mosaic virus	YP_009162614.2	*Umbravirus*
Patrinia mild mottle virus	YP_010087617.1	*Umbravirus*
Pea enation mosaic virus 2	NP_620846.3	*Umbravirus*
Tobacco bushy top virus	NP_733848.2	*Umbravirus*
Tobacco mottle virus	YP_009665185.1	*Umbravirus*
Angelonia flower break virus	YP_459960.2	*Alphacarmovirus*
Carnation mottle virus	YP_009032645.1	*Alphacarmovirus*
Tobacco necrosis virus A	NP_056825.2	*Alphanecrovirus*
Olive mild mosaic virus	YP_224015.1	*Alphanecrovirus*
Pothos latent virus	YP_009032634.1	*Aureusvirus*
Cucumber leaf spot virus	YP_009032639.1	*Aureusvirus*
Oat chlorotic stunt virus	NP_619751.1	*Avenavirus*
Turnip crinkle virus	NP_620720.3	*Betacarmovirus*
Cardamine chlorotic fleck virus	NP_041884.1	*Betacarmovirus*
Tobacco necrosis virus D	NP_608311.1	*Betanecrovirus*
Beet black scorch virus	NP_758810.3	*Betanecrovirus*
Galinsoga mosaic virus	NP_044732.1	*Gallantivirus*
Melon necrotic spot virus	NP_041226.1	*Gammacarmovirus*
Pea stem necrosis virus	NP_862835.2	*Gammacarmovirus*
Furcraea necrotic streak virus	YP_007517174.1	*Macanavirus*
Maize chlorotic mottle virus	NP_619718.1	*Machlomovirus*
Panicum mosaic virus	NP_068342.1	*Panicovirus*
Thin paspalum asymptomatic virus	YP_008219057.1	*Panicovirus*
Pelargonium ringspot virus	YP_009116639.1	*Pelarspovirus*
Pelargonium chlorotic ring pattern virus	YP_052925.1	*Pelarspovirus*
Cucumber necrosis virus	NP_040953.2	*Tombusvirus*
Tomato bushy stunt virus	NP_062897.1	*Tombusvirus*
Maize necrotic streak virus	YP_459920.2	*Zeavirus*
Carnation ringspot virus	NP_619711.1	*Dianthovirus*
Sweet clover necrotic mosaic virus	NP_620674.3	*Dianthovirus*
Barley yellow dwarf virus-MAV	NP_620064.1	*Luteovirus*
Barley yellow dwarf virus-PAS	NP_037635.1	*Luteovirus*
Barley yellow dwarf virus-PAV	NP_840014.2	*Luteovirus*
Pea enation mosaic virus 1	NP_620026.3	*Enamovirus*
Potato leafroll virus	NP_056748.3	*Polerovirus*

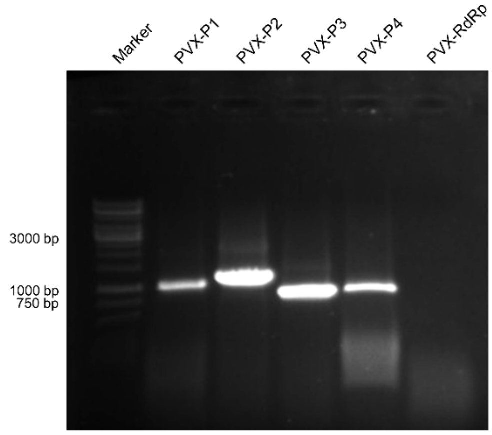

Figure S1. RT-PCR detecting PSCYV *P1, P2, P3, P4* and *RdRp* genes in the PVX-P1-, PVX-P2-, PVX-P3-, PVX-P4- and PVX-RdRp-infected plants at 21 dpi.
